# Immunohistochemical Detection of a Unique Protein within Cells of Snakes Having Inclusion Body Disease, a World-Wide Disease Seen in Members of the Families Boidae and Pythonidae

**DOI:** 10.1371/journal.pone.0082916

**Published:** 2013-12-10

**Authors:** Li-Wen Chang, Ann Fu, Edward Wozniak, Marjorie Chow, Diane G. Duke, Linda Green, Karen Kelley, Jorge A. Hernandez, Elliott R. Jacobson

**Affiliations:** 1 Department of Small Animal Clinical Sciences, College of Veterinary Medicine, University of Florida, Gainesville, Florida, United States of America; 2 Molecular Pathology Core, Department of Pathology, College of Medicine, University of Florida, Gainesville, Florida, United States of America; 3 Texas Department of State Health Services, Public Health Regions 8, Zoonosis Control Unit, Uvalde, Texas, United States of America; 4 Protein Biomarkers Laboratory, Proteomic Division, Interdisciplinary Center for Biotechnology Research, University of Florida, Gainesville, Florida, United States of America; 5 Hybridoma Laboratory, Cellomic Division, Interdisciplinary Center for Biotechnology Research, University of Florida, Gainesville, Florida, United States of America; 6 Electron Microscopy and Bio-imaging Core Laboratory, Cellomic Division, Interdisciplinary Center for Biotechnology Research, University of Florida, Gainesville, Florida, United States of America; 7 Department of Large Animal Clinical Sciences, College of Veterinary Medicine, University of Florida, Gainesville, Florida, United States of America; University of Georgia, United States of America

## Abstract

Inclusion body disease (IBD) is a worldwide disease in captive boa constrictors (*boa constrictor*) and occasionally in other snakes of the families Boidae and Pythonidae. The exact causative agent(s) and pathogenesis are not yet fully understood. Currently, diagnosis of IBD is based on the light microscopic identification of eosinophilic intracytoplasmic inclusion bodies in hematoxylin and eosin stained tissues or blood smears. An antigenically unique 68 KDa protein was identified within the IBD inclusion bodies, called IBD protein. A validated immuno-based ante-mortem diagnostic test is needed for screening snakes that are at risk of having IBD. In this study, despite difficulties in solubilizing semi-purified inclusion bodies, utilizing hybridoma technology a mouse anti-IBD protein monoclonal antibody (MAB) was produced. The antigenic specificity of the antibody was confirmed and validated by western blots, enzyme-linked immunosorbent assay, immuno-transmission electron microscopy, and immunohistochemical staining. Paraffin embedded tissues of IBD positive and negative boa constrictors (n=94) collected from 1990 to 2011 were tested with immunohistochemical staining. In boa constrictors, the anti-IBDP MAB had a sensitivity of 83% and specificity of 100% in detecting IBD. The antibody also cross-reacted with IBD inclusion bodies in carpet pythons (*Morelia spilota*) and a ball python (*python regius*). This validated antibody can serve as a tool for the development of ante-mortem immunodiagnostic tests for IBD.

## Introduction

Inclusion Body Disease (IBD) is a unique and insidious worldwide disease commonly seen in captive members of snakes in the families Boidae and Pythonidae [1]. The disease was most commonly reported in boa constrictors (*Boa constrictor*) and occasionally in other related snakes [1-14]. Although several retroviruses [2,9-11,13], and more recently, arena-like viruses have been identified in snakes with IBD [8,13,14], transmission studies using purified virus on live snakes will be needed to establish a causal relationship. Inclusion body disease is characterized by observation of eosinophilic intracytoplasmic inclusion bodies in hematoxylin and eosin (H&E) stained histological slides or blood smears, which is the current gold standard for diagnosing IBD [1,12]. Inclusion bodies seen in IBD sometimes can be difficult to distinguish from other cellular proteinaceus material or cellular granules that may accumulate in the cytoplasm of affected cells [1]. In some cases, the inclusion bodies may not be abundant in visceral tissue, or early developing smaller inclusion bodies may be overlooked in an H&E stained section. A validated molecular diagnostic test that is more sensitive and specific is needed.

Immunohistochemical (IHC) staining is a well recommended method with high sensitivity and specificity for diagnosing both infectious and non-infectious diseases [15]. With IHC staining, an antibody reacting to a specific antigen can be used to localize the antigen within affected tissue, which increases the accuracy of a diagnosis [15]. An antigenically distinct protein was identified within the characteristic intracytoplasmic inclusion bodies of boa constrictors [10]. The protein was approximately 68 KDa in molecular weight, and named “inclusion body disease protein” (IBDP) [10]. A monoclonal antibody (MAB) produced against IBDP reacted to the inclusion bodies in frozen tissue sections using IHC staining. Unfortunately, the antibody did not react to inclusion bodies in paraffin embedded tissues, and several years later the original hybridoma clone was lost (Wozniak, personal communication). Recently, polyclonal antibodies were produced against the predicted nucleoprotein of an arena-like virus and the isolated whole arena-like viruses, and these polyclonal antibodies recognized the IBD inclusion bodies [8,14]. However, the sensitivity and specificity of the polyclonal antibodies can vary from batch to batch, and these antibodies have not been validated for use in clinical diagnosis. An antibody recognizing IBDP can serve as a powerful tool for diagnosing IBD using IHC staining, and can be used to develop other immunobased diagnostic tests for IBD. 

For IHC staining in veterinary medicine, there is a lack of high quality antibodies that are made to identify specific antigens among different species [15]. This problem can be more significant for diagnosing diseases in zoological and exotic animal medicine where more diverse species are seen. Further, there appears to be a lack of standardization in IHC staining methodology among different veterinary laboratories [15]. The goal of standardization in IHC staining is to achieve reproducible and consistent results within and among different laboratories. Therefore, a MAB needs to be properly validated before being used or offered as a diagnostic reagent. 

In this study, we presented a modified method for isolating IBD inclusion bodies from crude tissue homogenates. An anti-IBDP MAB was produced against the semi-purified IBD inclusion bodies isolated from a boa constrictor, and the reactivity was confirmed by western blots, enzyme-linked immunosorbent assay (ELISA), immuno-transmission electron microscopy (immuno-TEM), and IHC staining on paraffin embedded tissues. Based on the guidelines suggested by Ramos-Vara et al. [15], we first determined the standard condition for IHC staining using the anti-IBDP MAB. Next, we validated the performance of the MAB by evaluating factors that may affect the IHC staining, which included the fixation time in formalin and the storage time in paraffin. Further, the cross-reactivity of this MAB produced for the use in boa constrictors was tested for its efficacy in reacting with inclusion bodies of other snake species having IBD, and other inclusion body forming diseases in snakes that appeared similar to IBD. Finally, the diagnostic performance of the IHC test using anti-IBDP MAB was determined.

## Materials and Methods

### Ethics Statement

 The collection of fresh and formalin fixed tissue samples, euthanasia of snakes, and the production of monoclonal antibody was authorized under University of Florida Institutional Animal Care and Use Committee Protocol 201101156.

### Sample Collection

A total of 112 snakes were used in this study, including 94 boa constrictors, 4 annulated tree boas (*Corallus annulatus*), 4 ball pythons (*Python regius*), 2 carpet pythons (*Morelia spilota*), 2 corn snakes (*Pantherophis guttatus*), 1 emerald tree boa (*Corallus caninus*), 4 rainbow boas (*Epicrates cenchria*), and 1 palm viper (*Bothriechis marchi*) (Table 1). Paraffin embedded tissues of 99 snakes were collected from the repositories of Anatomic Pathology Service and Zoological Medicine Infectious Disease Testing Laboratory, College of Veterinary Medicine, University of Florida (UF)(Gainesville, FL, USA), and additional blocks were kindly provided by Northwest ZooPath (Monroe, WA, USA) and Zoo/Exotic Pathology Services (West Sacramento, CA, USA). Fresh or formalin fixed tissue samples of 13 boa constrictors were collected throughout 2008 to 2011 by veterinarians in private practices or UF Veterinary Hospitals, and transferred to Zoological Medicine Infectious Disease Testing Laboratory, College of Veterinary Medicine, UF for IBD diagnosis. Portions of fresh tissues were fixed for processing into paraffin embedded blocks and immuno-TEM labeling, and the remaining biological materials were stored in an ultra-freezer at -80°C. A pathology report was available for each case with the final diagnosis verified by board certified pathologists based on histological examination of H&E stained sections. For each case, one block with standard tissues (liver, kidney, or pancreas) was selected for IHC staining. If IBD inclusion bodies were not described in the standard tissues, an additional block with tissues having IBD inclusion bodies was stained with IHC. 

**Table 1:** Summary of the collected samples. Diagnosis of IBD was based on the histological finding in H&E stained tissue sections.

**Sample Type**	**Year collected**	**Source of samples**	**Snake species**	**Diagnosis**	**n=**
Paraffin embedded tissue blocks	1990-2007	Anatomic Pathology Service, College of Veterinary Medicine, UF	Boa constrictors	IBD+	29
				IBD-	17
			Ball python	IBD+	2
			Carpet python	IBD+	1
			Corn snakes	IBD-	2
			Emerald tree boa	IBD+	1
			Rainbow boas	IBD+	4
	1990-2007	Zoological Medicine Infectious Disease Testing Laboratory, College of Veterinary Medicine, UF	Boa constrictors	IBD+	21
				IBD-	12
	2008-2011	Northwest ZooPath	Ball pythons	IBD+	2
			Carpet python	IBD+	1
			Palm viper	IBD+	1
	2008-2011	Zoo/Exotic Pathology Services	Annulated tree boas	IBD+	3
				IBD-	1
			Boa constrictors	IBD+	2
Fresh or formalin fixed tissues	2008-2011	Zoological Medicine Infectious Disease Testing Laboratory, College of Veterinary Medicine, UF	Boa constrictors	IBD+^a^	8
				IBD-^b^	5

^a^ Of 8 IBD+ samples collected from boa constrictors, 5 were submitted as formalin fixed tissues and the other 3 were submitted as fresh tissues on ice. For each sample, portions of the fresh tissues were fixed in 10% NBF for processing into paraffin embedded blocks, and another portion was fixed and embedded for immuno-TEM. The remaining materials were stored at -80°C. Tissues that were submitted as formalin fixed tissues were processed into paraffin blocks for the use in this study.

^b^ Of 5 IBD- samples collected from boa constrictors, 3 were submitted as formalin fixed tissues and 2 were submitted as fresh tissues on ice. For each sample, portions of the fresh tissues were fixed in 10% NBF for processing into paraffin embedded blocks. The remaining materials were stored at -80°C. Tissues that were submitted as formalin fixed tissues were processed into paraffin blocks for use in this study.

### Purification of IBDP

The purification method was modified from a published protocol for isolating human Mallory bodies [16]. Briefly, approximately 5 g of frozen liver and kidney obtained from a boa constrictor were thawed and separately processed. Each tissue was cut into small portions (approximately 3 mm cubes) and homogenized in homogenization buffer (HB) containing, 250 mM sucrose, 10 mM EDTA, 10 mM HEPES, pH 7.4. The tissue to be homogenized was processed 1-2 g at a time with 5 mL HB, and the homogenate was collected and filtered through two layers of gauze, to remove the larger tissue debris. The homogenate was centrifuged at 1000 x g for 10 minutes. The supernatant was removed and the soft pellets were pooled and re-suspended with equal volume of HB, mixed with 1% Sarkosyl (Teknova, 2S3380) at a 1:1 ratio and incubated in 37°C for 30 minutes with frequent vortexing. The suspension was centrifuged at 14,000 x g for 10 minutes at 4°C, and the supernatant was removed. The pellet was resuspended with equal volume of 1% sarkosyl, and the suspension was subjected to one more round of the above procedure, with incubation followed by centrifugation. After removing the supernatant, the pellet was carefully re-suspended in 1 mL of HB which will be referred to hereafter as “inclusion body preparation” (IB prep). The IB preps were stored at 4°C for future analysis.

### Cytospin Preparation

Suspensions collected from each protein purification step were diluted 50 fold with water, and 50 µL of each diluted sample was placed in a cytocentrifuge chamber (Biomedical Polymers Inc., BMP-CYTO-S50), and centrifuged for 6 minutes at 800 rpm onto a glass microscopic slide using a Cytospin centrifuge (Shandon, Cytospin 2). The slides were air dried, fixed in 10% neutral buffered formalin (10% NBF) (Fisher Scientific, SF100-20) for 10 minutes, washed with distilled water, and stained with H&E stain.

### Electrophoresis and Gel Staining

Aliquots of IB preps or crude tissue homogenates were reduced by addition of equal volume of 2X loading sample buffer (25 mM Tris, 4% SDS, 100mM DTT, and 30% glycerol). For western blots, the proteins were reduced by addition of 4X NuPAGE LDS sample buffer (Invitrogen, NP0008) and 10X NuPAGE reducing agent (Invitrogen, NP0004) with 500 mM DTT. Following addition of the reducing buffer, the samples were heated to 95-100 °C for 10 minutes. Next, the total protein concentration of a 1:10 dilution of each reduced and unreduced sample was estimated by standard Bradford Protein Assay (Bio-Rad Laboratories, Inc.), subsequently, the total protein concentration of the IB preps were estimated. The reduced proteins were resolved on a 10% or 4%~12% NuPAGE Bis-Tris gel (Invitrogen) with MES or MOPS buffer (Novex) at 200V constant voltage. For evaluation of protein quality, 5 µg of protein were loaded in each well, and the resolved protein was visualized by SimplyBlue (Novex) stain, using the microwave protocol provided by the manufacturer (Novex).

### Electro-elution of IBDP

The resolved 68 KDa IBDP bands were cut from the gels, minced, and packed inside glass tubes of the Model 422 Electro-eluter (Bio-Rad, 165-2976). The protein was electro-eluted in Tris-Glycine buffer without SDS, following the protocol provided by the manufacturer (Bio-Rad).

### Protein Solubilization

Several solubilizing reagents were used alone or in combination in an attempt to solubilize the isolated insoluble inclusion bodies (IB preps). The tested solubilizing reagents included, 8-12 M urea, 6 M guanidine hydrochloride (Gu-HCl), 1% Triton-100, 2% octyl beta-glucoside (OBG), 1% dodecyl maltoside (DDM), 2-4% SDS, 20% lithium dodecyl-sulfate (LDS), 1 M DTT, dimethyl sulfoxide (DMSO), bicarbonate buffer and 1% acetic acid. Additionally, a combination of 1 M DTT with 8 M urea, or 6 M Gu-HCl, or 1% Triton-100, or 2% OBG, or 1% DDM, or 20% LDS, or 4% SDS were tested. Thirty microliters of IB prep was placed in a 1.5 mL tube, and centrifuged at 12,000 rpm (15,294 x g) for 20 minutes at 4°C using an Eppendorf 15 Amp Centrifuge Model 5810R (Eppendorf North America, Hauppauge, New York). After centrifugation, the supernatant was removed, and the pellet was resuspended in 30 µL of a solubilizing reagent. For the use of combined reagents, 30 µL of DTT was added into the tube. After thorough vortexing, the tube was maintained at room temperature (RT) for 30 minutes, followed by centrifugation again at 12,000 rpm for 20 minutes. Solubilization was monitored by visually comparing the size of the pellet in the treated sample with that in an untreated sample. If the inclusion bodies were partially solubilized by the reagent, the pellet would appear smaller than the pellet of the untreated sample. If the inclusion bodies were completely solubilized, no pellet would be observed after centrifugation.

### Polyclonal and Monoclonal Anti-IBDP Antibody Production

Mouse monoclonal antibody against IBDP was produced using the standard protocol of the Hybridoma and Protein Core Laboratories, UF Interdisciplinary Center for Biotechnology Research (ICBR) [17]. Modifications were necessary due to the insolubility of IBDP. Briefly, two female Balb/cByJ mice were immunized with approximately 100 µg of isolated inclusion bodies (IB prep of #08-76) diluted in sterile physiologic phosphate buffered saline (PBS) and emulsified in Ribi MPL+TDM adjuvant. The immunogen was administered on day 1, 21, 44, and 192. The test bleeds were collected 11 to 14 days after the second and third immunizations. The presence of anti-IBDP antibodies in the post-immunized serum was determined by western blots, ELISA, and IHC staining. Six days after the fourth immunization, mouse 1 was euthanized and the splenic lymphocytes were collected and fused with mouse myeloma cells to form hybridoma cells [17]. The cultured media of the growing hybridoma mass cultures were collected and screened for anti-IBDP antibody production by ELISA. The mass cultures that tested positive by ELISA were subsequently tested by western blots. The mass cultures that had the highest direct optical density (OD) reading on 3 or 4 different IB preps were further tested for their reactivity to the 68 KDa IBDP by western blots. The cultures that showed reactivity to IBDP in both ELISA and western blots were grown out, and further cloned by limiting dilution. The cultured media collected from each clone were tested again by ELISA, western blots and IHC staining. The monoclonal antibodies were isotyped by ELISA and IsoStrip test following manufacturer’s protocol. The cultured medium of the final selected hybridoma clone was harvested, and purified through a protein G column (GE Healthcare Protein G Sepharose 4 Fast Flow). The concentration of the purified monoclonal anti-IBDP antibody was determined by Bradford Protein Assay and stored at 4°C for future validation.

### Western Blots

The resolved protein gel was transblotted onto a nitrocellulose membrane using standard protocol of the iBlot dry blotting system (Invitrogen). The membrane was blocked, washed, incubated, and developed following previously described method [17]. Briefly, after blocking and washing, each lane of the membrane was separated by a Fast Blot-Developer manifold (Pierce, 88040), and incubated with mouse serum in a dilution of 1:100 or 1:500 for 1 hour. For hybridoma mass culture and clone screening, each lane was incubated with undiluted cultured medium. The blot was washed, and incubated with alkaline phosphatase-conjugated rabbit-anti-mouse antibody (Sigma, A1902) at a dilution of 1:1,000 for one hour. After washing, the blot was colorometrically developed by incubation with BCIP/NBT alkaline phosphatase substrate (Sigma, B5655) following instructions of the manufacturer.

### ELISA

Flat bottom 96 well assay plates were coated with IB prep that was diluted in optimal concentrations (10, 20, 30, 40 µg/mL) with bicarbonate buffer. The IB preps isolated from liver and kidney of 2 IBD positive boa constrictors (#08-76, #08-122) were used as coating antigens on separate plates. The general ELISA procedure was the standard protocol used in Hybridoma and Protein Core Laboratories, UF ICBR [17]. Briefly, the plates were coated by incubation with IBDP overnight at 4°C, washed, blocked, and incubated with diluted mouse serum or undiluted cultured medium of the hybridoma cells, and incubated for 1 hour at RT. After washing, the wells were incubated with a 1:1000 dilution of alkaline phosphate conjugated rabbit anti-mouse IgG antibody (Sigma, A1902) for 1 hour at RT. For antibody subtyping, a conjugated anti-mouse IgG antibody (Sigma, A3438) or a conjugated anti-mouse IgM antibody (Sigma A9688) were used as the secondary antibodies. Subsequently, the wells were washed again and developed with para-nitrophenyl phosphate substrate (PnPP; Sigma, N2765) for 1 hour at RT. The direct OD values of each well were recorded with the absorbance at 405 nm, and compared against the baseline reading, which were the OD reads of wells coated with fresh cell culture medium.

### Immuno-Transmission Electron Microscopy

Fresh liver and kidney tissues collected from three IBD+ boa constrictors were submitted for examination using TEM. The tissues were fixed with electron microscopy grade 4% paraformaldehyde (4% PF), 1% glutaraldehyde in 1X PBS, pH 7.24. The samples were washed in PBS pH 7.24, subsequently water washed and dehydrated in a graded ethanol series (25%, 50%, 75%, 95%, 100%, 100%), infiltrated in Lowicryl HM20 acrylic resin (Electron Microscopy Sciences, Hatfield, PA, USA) and UV cured at -10°C for 48 hours. Cured resin blocks were trimmed, thin sectioned and collected on Formvar coated Ni 400 mesh grids (Electron Microscopy Sciences, Hatfield, PA, USA). Ultrathin sections were immuno-labeled at RT as follows; the grids were treated with 200 mM NH_4_Cl in high salt Tween-20 (HST) for 20 minutes, rinsed in HST, incubated 1 hour with blocking solution (1.5% BSA, 0.5% cold water fish skin gelatin, 0.01% Tween-20 in HST, pH 7.2), and incubated with immunized mouse 1 serum in 1:100 dilution, or the purified anti-IBDP MAB in 1:10 dilution overnight at 4°C. For negative controls, the grids were incubated with non-related mouse IgG antibodies (ICBR-EMBL private stock). The following day, the grids were washed three times in PBS, and incubated for 1 hour at 21°C on 18 nm colloidal gold affinity-purified goat anti-mouse IgG (Jackson ImmunoResearch, West Grove, PA, USA) diluted 1:30 in PBS. Subsequent washes in PBS and distilled water, post-stained with 2% aq. uranyl acetate and Reynold’s lead citrate. Sections were examined with a Hitachi H-7000 TEM (Hitachi High Technologies America, Inc. Schaumburg, IL, USA) and 2k x 2k digital images acquired with a Veleta camera and iTEM software (Olympus Soft-Imaging Solutions Corp, Lakewood, CO, USA).

### Formalin Fixation and Embedding

Fresh tissues including liver, kidney, and pancreas obtained from five euthanized boa constrictors were dissected into approximately 5 mm thick sections, placed in cassettes, fixed in 10% NBF or 4% PF for 48 hours, and finally embedded into paraffin. In order to evaluate the effects of formalin fixation time, freshly obtained liver, kidney, and pancreas of an IBD+ boa constrictor were each cut into 10 sections that were approximately 5 mm thick. One piece of each sectioned liver, kidney, and pancreas were placed in a cassette (ten sets of tissues), followed by fixation in ten identical containers filled with 10% NBF. On Day 2 (48 hours after initial fixation), Day 7, Day 8, Day 9, Day 15, Day 23, Day 32, Day 39, Day 50, and Day 58, one cassette was removed and the tissues were embedded in paraffin. For tissue set of Day 58, only kidney and pancreas were embedded.

Paraffin embedded tissues obtained from the case repositories were embedded in the laboratory of Anatomic Pathology Service in the UF Veterinary Hospital (Lab 1). Using an automated processor (Thermo Electric Corporation, Shandon Excelsior), the fixed tissues were dehydrated in graded ethanol, followed by infiltration of xylene and paraffin. The processed tissues were manually mounted in paraffin blocks. In order to evaluate the IHC staining condition, liver and pancreas of one boa constrictor were fixed, processed, and embedded separately in two laboratories, Lab 1 and the laboratory of Molecular Pathology Core (Lab 2), College of Medicine, UF. The embedding procedures of the two labs can be found in Table S1. 

### H&E Staining

All H&E staining were done by an automatic slide stainer (Gemini Varistain, Thermo Shandon, Illinois, IL). For paraffin embedded tissues, the slides were deparaffinized with xylene, and the tissue sections were rehydrated in a graded series of ethanol solutions. The rehydrated tissues were stained with hemotoxylin (Richard-Allan Scientific, 7212) for 2 minutes, incubated with clarifier 2 (Richard-Allan Scientific, 7402) for 30 seconds, followed by incubating with bluing reagent (Richard-Allan Scientific, 7301) for 1 minute, then incubated one minute in 80% ethanol before staining with eosin (Richard-Allan Scientific, 71311) for 1 minute. In between the application of each reagent, the slides were washed with running water. For the cytospin prepared IB preps, the fixed microscopic slides were stained with the above protocol with minor modifications as follow. After staining with hemotoxylin, the slides were incubated with clarifier 2 for 15 seconds. After bluing, the slides were stained with eosin without incubating with 80% ethanol. Finally, the H&E stained slides were dehydrated in a graded ethanol series, dipped in xylene, and coverslipped.

### IHC Staining

Immunohistochemical staining was used for confirming the reactivity of the antibodies to the IBD inclusion bodies within tissue sections. The staining was performed in Lab 2 using the methods provided below. 

#### Slide preparation and antigen retrieval (AR)

For paraffin embedded tissues, the sections were deparaffinized in xylene, followed by rehydrating in graded ethanol, and finally rinsed with water. The deparaffined tissues were either treated with AR reagents or were not treated with AR reagents. The following AR reagents were evaluated: trypsin (Invitrogen, Digest-All2), Trilogy (Cell Marque), Citra (Biogenex), Dako Target Retrieval Solution, pH 6.0 (DAKO), Dako Target Retrieval Solution, pH 9.0 (DAKO). The trypsin AR was done by incubating the slides for 5 minutes at 37°C. The AR treatments with other reagents were done by incubating the slides for 30 minutes at 95°C. For double AR treatment, the slides were incubated with Trilogy for 30 minutes at 95°C, followed by additional 5 minutes of incubation with trypsin at 37°C. 

#### Primary antibody incubation

The prepared slides were washed with tris-buffered saline (TBS), and blocked with Sniper blocking reagent (Biocare Medical, BS966) for 15 minutes at RT. After washing again with TBS, the blocked tissues were covered with diluted mouse serum in antibody diluent (Invitrogen, 00-3218) or with medium collected from the growing hybridoma clones, and incubated overnight at 4°C. For antibody validation, the slides were covered by anti-IBDP MAB in a specific dilution (1:1,000, 1:2,000, 1:5,000, 1:10,000, 1:20,000), and incubated for 1 hour at RT, or overnight at 4°C. 

#### Detection system and counter staining

After washing, the paraffin embedded tissues were incubated with 3% peroxide in methanol for 10 minutes. The slides were rinsed with water and washed by TBS before covering the tissue with HRP conjugated goat-anti-mouse antibody (Biocare Medical, MHRP520) for 30 minutes at RT. After washing, the HRP-conjugated secondary antibody was visualized by development with diaminobenzidine (DAB; Vector Laboratory, SK-4100) or VECTOR NovaRED (Vector Laboratory, SK-4800) according to the manufacturer’s protocol. For the standardized IHC staining, all slides were stained using NovaRED HRP chromagen. The tissues were counterstained with hematoxylin, dehydrated in graded ethanol, placed in xylene, and cover slipped by Cytoseal XYL (Thermo Scientific).

#### Automated staining machine

For the tissues to be stained automatically, after AR and blocking, the slides were applied onto the automated staining machine (Autostainer Plus, DAKO), in which the washes and incubation of the primary and secondary antibodies were performed. Subsequently, the slides were developed manually using the procedures described above. 

### IHC Evaluation

Using light microscopy, the intensity of the IHC stain was given one of the four scores: 0 (no staining), 1 (faint, barely visible), 2 (moderate), 3 (strong). For each IHC staining run, a positive control slide (IHC score 3) and negative control slides (IHC score 0) were stained parallel to the slides to be scored. The negative control of each sample was a duplicated slide that stained with a commercial non-specific mouse IgG instead of anti-IBDP antibody.

For evaluating the effect of storage time in paraffin and cross reactivity of the anti-IBDP MAB, the IHC staining results were interpreted as either IHC stain positive or negative. A positive IHC stain (IHC+) was defined by a visible staining pattern (IHC score 1 to 3) compared to the negative control (IHC score 0). A negative IHC stain (IHC-) was defined by no visible staining pattern (IHC score 0) compared to the negative control (IHC score 0).

### IHC Diagnostic Performance Evaluation and Statistics

In this study, samples with a positive or negative diagnosis for IBD by examination of the H&E stain (current gold standard) were classified as IBD+ or IBD-, respectively. The sensitivity, specificity, positive predictive value (PPV) and negative predictive values (NPV) of the IHC test, compared to H&E, were calculated following standard procedures [18]. Sensitivity was defined as the proportion of samples classified as H&E stain positive that tested positive by using the IHC test. Specificity was defined as the proportion of samples classified as H&E stain negative that tested negative by using the IHC test. Positive predictive value was defined as the probability that given a positive IHC test, the sample actually tested positive by using H&E stain. Negative predictive value was defined as the probability that given a negative IHC test result, the sample actually tested negative by using H&E stain. Positive and negative predictive values were calculated using prevalence estimates ranging from 5% to 80% with the calculated sensitivity and specificity of the IHC test determined by this study.

In order to assess the effect of storage time in paraffin, the paraffin embedded tissues were divided into two groups: Group 1. tissue samples embedded within the time period 1990-2000, and Group 2. tissue samples embedded within the time period 2001-2011. The sensitivity and specificity of the IHC test in Group 1 and Group 2 were compared using the Fisher Exact X^2^ test, following standard procedures [19].

## Results

### IBDP Purification

**Figure 1:**
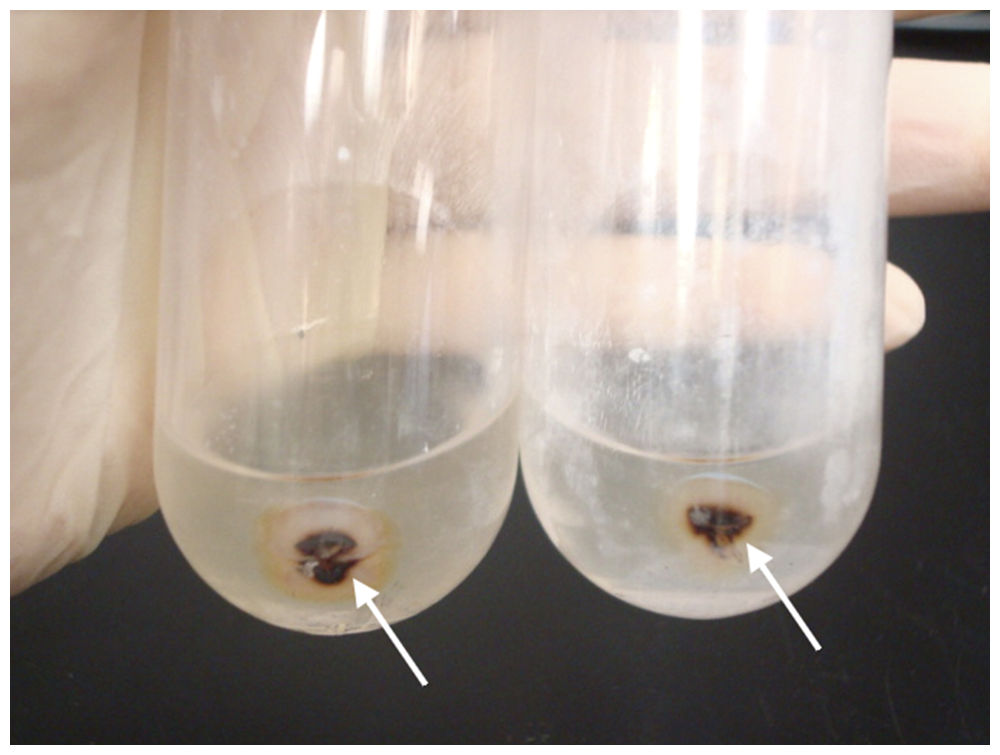
The isolated inclusion bodies obtained from 6 g of IBD+ liver. The arrows showing pelleted inclusion bodies in 1% Sarkosyl after centrifugation.

**Figure 2:**
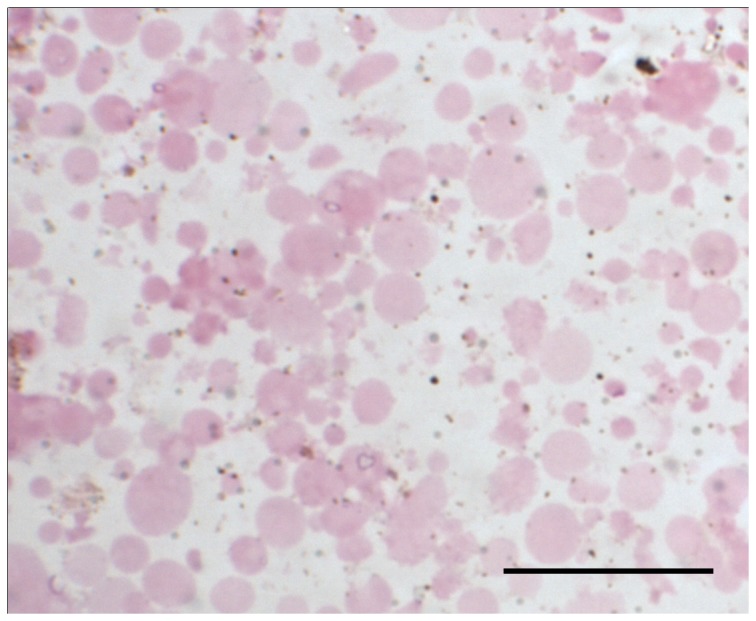
The H&E stained IB prep on a microscopic slide. The semi-purified inclusion bodies were used to immunize mice for antibody production. Bar = 20 µm.

**Figure 3:**
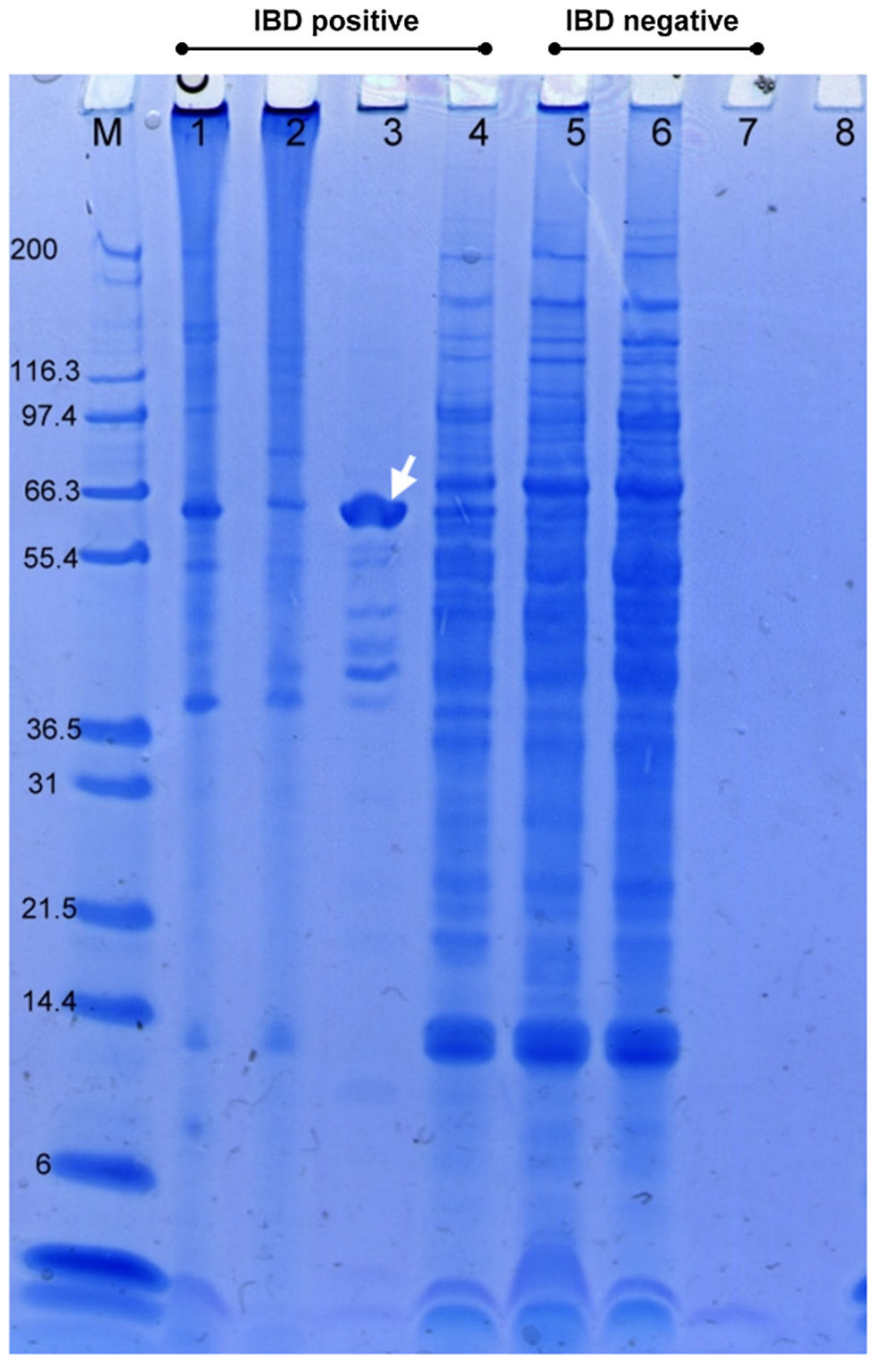
The resolved IB preps and liver homogenates on a NuPAGE. Ten microliters of protein were loaded on each lane. Lane 1 to 3 are three different IB preps obtained from three IBD positive boa constrictors. Lane 4 is the liver homogenate from the same boa as lane 3. Lane 5 and 6 are liver homogenates from two IBD negative boas. Lane 7 is the IB prep derived from an IBD negative boa, which no pellet were left after incubating with 1% sarkosyl. Lane 8 is HB served as a blank control. The IB prep from #08-76 showed a major intense band approximately at 68 KDa (arrow), showing that the IBDP is concentrated in the IB prep compared to the liver homogenate (Lane 4). M. Molecular weight marker (Invitrogen, Mark 12).

Inclusion body preps and total liver homogenates from 3 IBD+ and 2 IBD- boa constrictors were obtained. The IBD- liver did not result in any solid pellet after the incubation with sarkosyl, whereas a tightly bound pellet was obtained from all three IBD+ samples (Figure 1). Among the IB preps, the best quality samples were those that contained the most inclusion bodies and the least amount of extraneous cellular materials. The presence or absence of protein bands other than the 68 KDa band, and the intensity of the resolved 68 KDa protein band on a NuPAGE gel determined the quality of IB preps. Under light microscopy, the H&E stained IB prep from snake #08-76 appeared as a suspension of primarily eosinophilic globules of various sizes with minimal extraneous material (Figure 2). When resolved on the gel, this IB prep consisted of a major intense band with a molecular weight of slightly less than 68 KDa (Figure 3, lane 3). Despite the slight shift in molecular weight, possibly caused by the differences in the gel and electrophoresis buffer combination, this isolated protein was consistent with the previously described IBDP [10]. When resolved on a NuPAGE gel, the 68 KDa protein isolated from snake #08-76 through the inclusion body isolation procedure appeared to be highly concentrated compared to the respective crude liver homogenate (Figure 3, lane 4). 

All attempts to completely solubilize the IB prep using the common reducing and solubilizing agents were unsuccessful. Following centrifugation, the semi-purified inclusion bodies formed a tightly bound pellet that was difficult to resuspend. Small amounts of inclusion bodies were lost as a consequence of binding to the inner surface of plastic tubes and pipets. This was more prominent when placed in aqueous solutions (such as water, HB or PBS), than in solutions containing urea, Gu-HCl, OBG, and sarkosyl. Combination of 4% SDS or 12 M urea with 500 mM DTT could not completely solubilize IBDP. Prior to protein electrophoresis, the IB preps were reduced by sample buffers containing high concentration of solubilizing reagents (2% SDS or 2% LDS with 50-500 mM DTT) that were heated to 95-100 °C for 10 minutes. Still, a portion of inclusion bodies remained insoluble. The reduced IBDP that was resolved by electrophoresis became insoluble again when electro-eluted. The eluted IBDP coated the membrane of the gel eluter collecting chamber, and could not be retrieved. The solution collected from the collecting chamber had a protein concentration that was too low for antibody production. Due to the insolubility of the IB preps, further protein purification methods such as 2D-electrophresis and liquid chromatography were not feasible. Nevertheless, the IBDP (IB prep) from snake #08-76 was considered to be of sufficient purity to serve as the best immunogen available for anti-IBD MAB production.

Due to the insolubility of the IB preps, the IBDP could not be detected in the Bradford Protein Assay without reduction by heating up to 95-100°C for 10 minutes in 2% SDS and 50 mM DTT. However, with 10 fold dilution of the reduced sample, the concentration of the SDS (0.2%) still exceeded the tolerance of SDS level (0.025%) in Bradford Protein Assay. Thus, it was suspected that there was interference with the estimated protein concentration. Therefore, for all assays (western blots and ELISA) used in antibody selection, IB preps were quantified by the volume of the IB prep needed for a sufficient reaction in the assay determined by a test run prior to the actual assay, and the amount of protein equivalent to the volume was documented. 

### Antibody Production and Monoclonal Antibody Selection

Polyclonal antibody that was reactive to the 68 KDa protein band was detectable in the mouse serum by western blots and IHC on day 57 post-immunization. Cultured media of hybridoma mass cultures derived from splenic lymphocytes of mouse 1 were screened for reactivity to the IB preps by ELISA. Four different IB preps were used as coating antigens, including liver and kidney isolates from two IBD+ boa constrictors (#08-76 and #08-122). Each hybridoma mass culture was tested for the reactivity against 2 to 3 IB preps. Of 303 hybridoma mass cultures screened, only 1 culture (5B3) showed a low positive reactivity (OD reading approximately 5 folds higher than the baseline) and cross-reacted with 3 different IB preps. Additionally, 32 mass cultures that had significantly higher OD readings compared to the background were also selected. Collected cultured media of the 33 selected mass cultures were further tested for the reactivity to IB preps by western blots. Only antibodies produced by mass culture 5B3 showed reactivity to the 68 KDa protein band, and reacted to all 4 IB preps.

The mass culture 5B3 was further cloned by limiting dilution and seeded at a single cell per well density. Of 72 single colony wells that were screened for reactivity to IB prep by ELISA, 10 wells that showed low positive reactivity (OD read approximately 5 fold of the baseline) were selected, and further tested for their reactivity to all four IB preps. The monoclonal antibodies were isotyped and determined to be IgG subtype by ELISA. Seven out of the 10 selected clones were further tested for reactivity to the 68 KDa band of 4 IB preps by western blots, and for their IHC reactivity to the inclusion bodies in liver and pancreas of boa #08-76 and #08-122. All of the tested clones showed positive reactivity to the 68 KDa and inclusion bodies by western blot and IHC staining respectively. The clone 5B3-3D9 that showed less background in IHC staining was selected and grown to high density for subsequent purification. From 120 mL of cultured medium that was harvested, and purified, a total yield of 16.9 mg anti-IBDP monoclonal antibody at a concentration of 10.37 mg/mL was obtained. This antibody was isotyped as IgG1 with kappa light chains by IsoStrip.

### Immuno-Transmission Electron Microscopy

**Figure 4:**
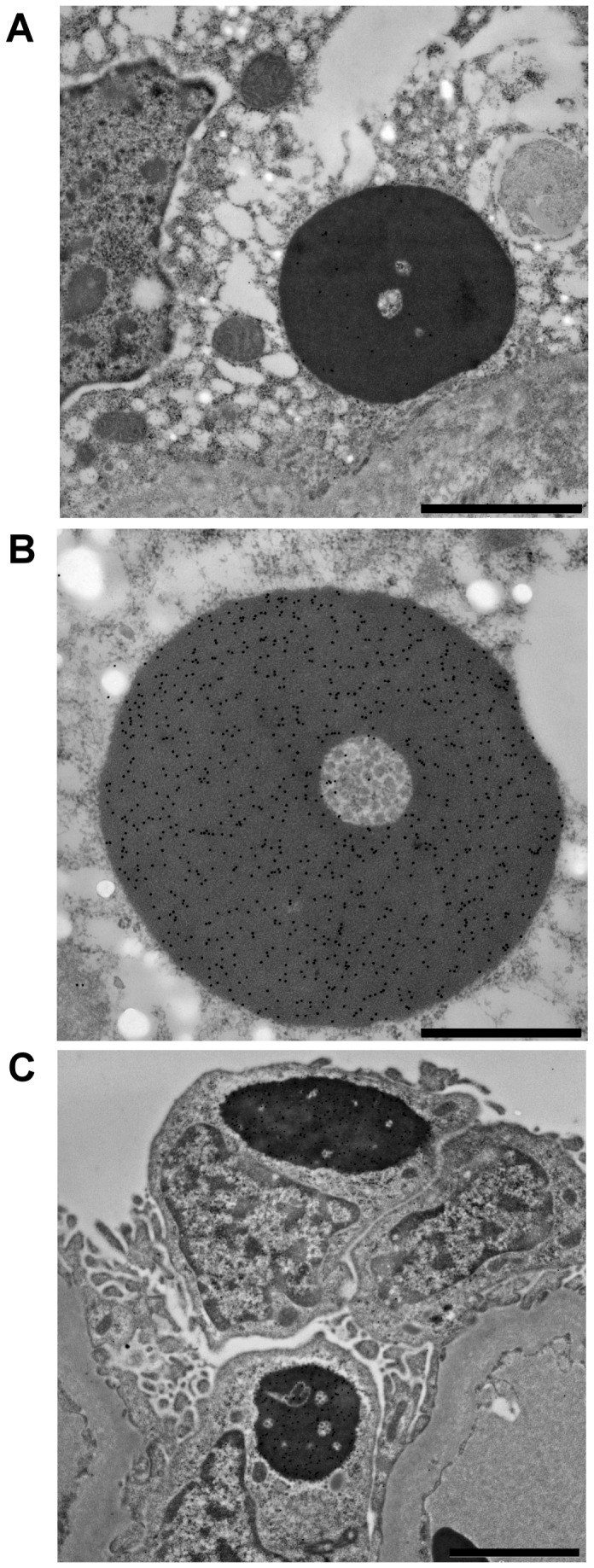
Immuno-gold labeling of IBD affected liver and kidney from boa constrictors. A. gold particles conjugated with purified anti-IBDP MAB labeling an inclusion body within liver of a boa constrictor. Bar = 2 µm. B. gold particles conjugated with polyclonal antibody (Mouse 1 serum) labeling an inclusion body within kidney of a boa constrictor. Bar = 1 µm. C. gold particles conjugated with polyclonal antibody (Mouse 1 serum) labeling an inclusion bodies within kidney of a boa constrictor. Bar = 2 µm.

Under TEM, the inclusion bodies within liver and kidney of 3 IBD+ boa constrictors were detected by mouse 1 serum or anti-IBDP MAB, which were labeled with conjugated gold particles (Figure 4). The gold particles specifically labeled the inclusion bodies, with no to minimal non-specific labeling of other cytoplasmic or nuclear structures. This demonstrated that the antibodies were highly specific to IBDP.

### Standardization of IHC Staining Condition

In order to establish a consistent staining performance, we initially used the paraffin embedded tissues of two boa constrictors for IHC staining to determine the ultimate staining condition for our system using the anti-IBDP MAB. The criteria for standardization were based on the suggested guidelines by Ramos-Vara et al. [15], which included species for testing, tissue type, fixation method, AR, incubation conditions of the antibody, and the detection system. Due to the availability of materials, boa constrictors were selected as the standard species. Liver, kidney, and pancreas were selected as the standard tissues for IHC staining. 

#### Fixation method

We found that tissues fixed in 4% PF stained adequately (IHC score 3) with only mild AR treatment or no treatment at all. Tissues that were fixed in 10% NBF sometimes required AR treatment for adequate IHC staining intensity (IHC score greater than 0 or 1). However, considering that 10% NBF was most commonly used in routine histopathological evaluation, we selected it as the standard fixation method.

#### Antigen retrieval

A variety of AR reagents including, trypsin, Trilogy, Citra, Dako Target Retrieval solution pH 6.0, and Dako Target Retrieval solution pH 9.0 were tested to determine which reagent resulted in the best staining intensity in IHC staining on the selected tissues fixed with 10% NBF. When trypsin was used, the blocks made in Lab 2 stained strongly with MAB (IHC score 3), but the blocks made in Lab1 stained faintly (IHC score 1) or no staining (IHC score 0). Using harsher AR treatment such as, Trilogy, Citra, Dako Target Retrieval solution (pH 6.0 and pH 9.0), the staining intensity was improved to medium or high (IHC score 2 to 3) (Table S2). Trilogy was finally selected as the standard AR reagent, because it generated high staining intensity and less non-specific background staining compared to the other reagents tested.

#### Primary antibody

The standard dilution for the primary antibody was determined by testing anti-IBDP MAB in a series of dilutions until the IHC staining intensity started to decrease. The staining intensity remained high with very minimal background in a dilution of 1:10,000, but the staining intensity decreased in a dilution of 1:20,000. Thus, the dilution of 1:10,000 was determined as the standard dilution for the anti-IBDP MAB. The incubation time of the primary antibody showed no significant differences in the staining intensity between 1 hour incubation at RT and overnight incubation at 4°C. Therefore 1 hr was used as the standard incubation time. Since there was no significant difference in the IHC staining intensity between manually and automated staining methods, the later method was selected as the standard protocol. This would enhance ‘run to run’ and ‘inter-run’ consistency. 

#### Detection system

**Figure 5:**
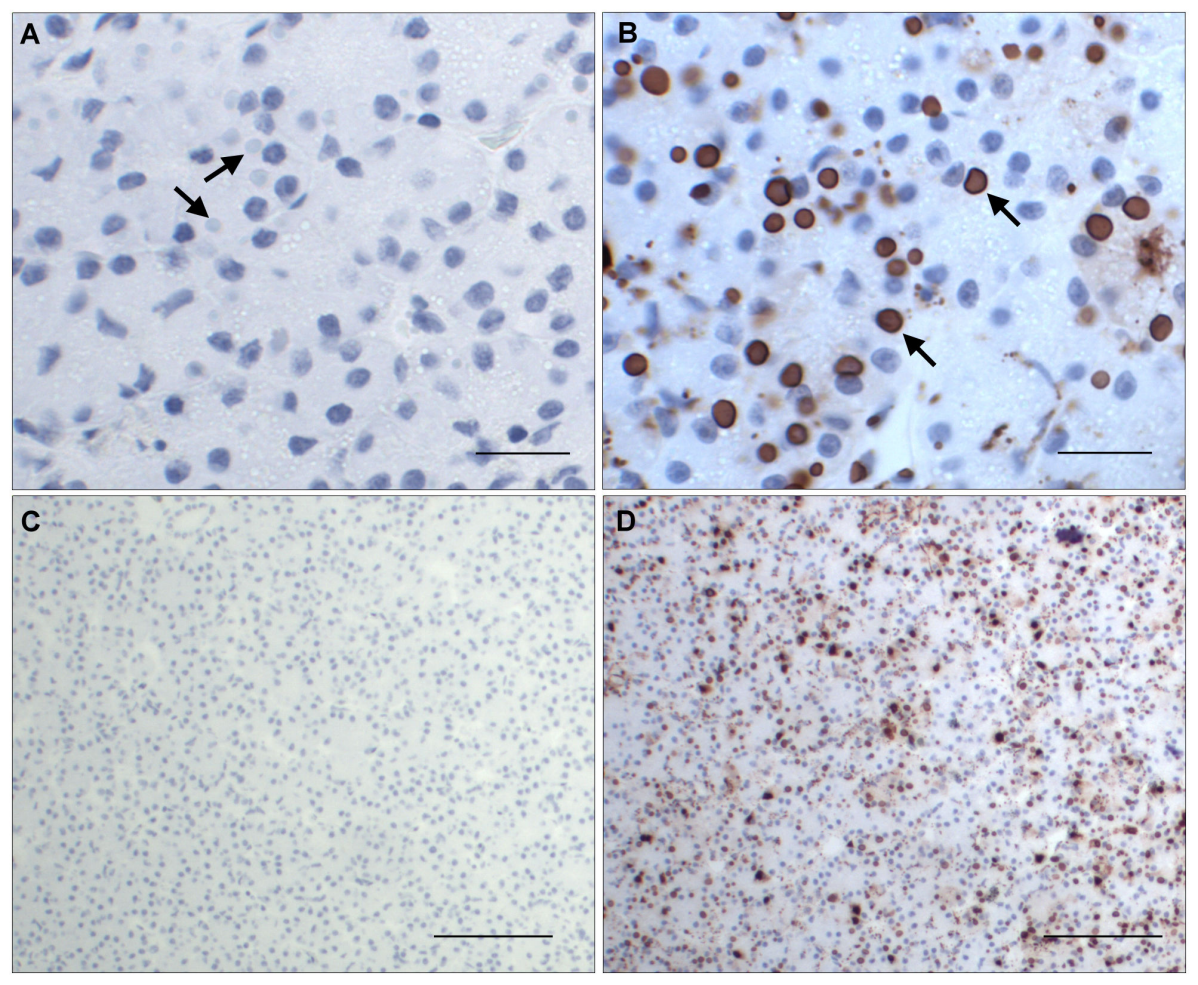
Pancreas of an IBD positive boa constrictor stained with standardized IHC condition. The tissue was fixed and stained under standardized IHC staining conditions using NovaRED as substrate. The negative control was stained with non-specific mouse antibody. The cell nucleus stained dark blue with hematoxylin, and the inclusion bodies are indicated by arrows. A. The inclusion bodies were not stained in the negative control slide. Bar = 20 µm.. B. The inclusion bodies stained dark red by anti-IBDP MAB. Bar = 20 µm. C. The inclusion bodies were not stained in the negative control slide. Bar = 100 µm. D. The positive stained pancreas by anti-IBDP MAB. Bar = 100 µm.

The chromogenic peroxidase detection system was used throughout all IHC staining, with HRP conjugated goat-anti-mouse antibody detected by DAB or NovaRED. The substrate DAB stained reactive sites with a brown color that sometimes could be confused with the brown pigments of the hepatic melanomacrophages in liver tissues. The substrate NovaRED stained the reactive sites with a reddish-purple color that contrasted better than DAB in the bluish hemotoxylin stained background (Figure 5). Therefore, NovaRED was selected as the standardized detection system.

Finally, the IHC staining conditions for the use of anti-IBDP MAB were standardized and summarized in Table 2.

**Table 2:** Standardized IHC staining conditions for anti-IBDP MAB.

**Key steps in IHC stain**	**Standardized conditions**
Species	*Boa constrictor*
Tissue type	Liver, kidney, pancreas
Fixative	10% NBF
Fixation time	48 hours
Antigen retrieval	Triology (95°C 30 minutes)
Primary antibody*	Anti-IBDP MAB (1:10,000 dilution)
Incubation time*	1 hour RT
Secondary antibody*	HRP conjugated goat-anti-mouse antibody (MACH 2 Mouse HRP-Polymer, Biocare Medical)
Incubation time*	30 minutes RT
Detection system	Chromogenic peroxidase with NovaRed substrate
Counter staining	Hematoxylin (blue)

* For standardized IHC staining of this study, these steps were performed in automated staining machine.

### Validation of Anti-IBDP MAB with IHC Staining

According to the IHC guidelines [15], the validation of the anti-IBDP MAB was performed using the standardized IHC staining conditions, and the performance of the IHC test using this antibody was determined. The factors that may affect the IHC staining were evaluated, which included the fixation time in formalin, the storage time in paraffin, the cross-reactivity with IBD inclusion bodies of other snake species and other inclusion body forming antigens. 

#### Effects of formalin fixation time

**Figure 6:**
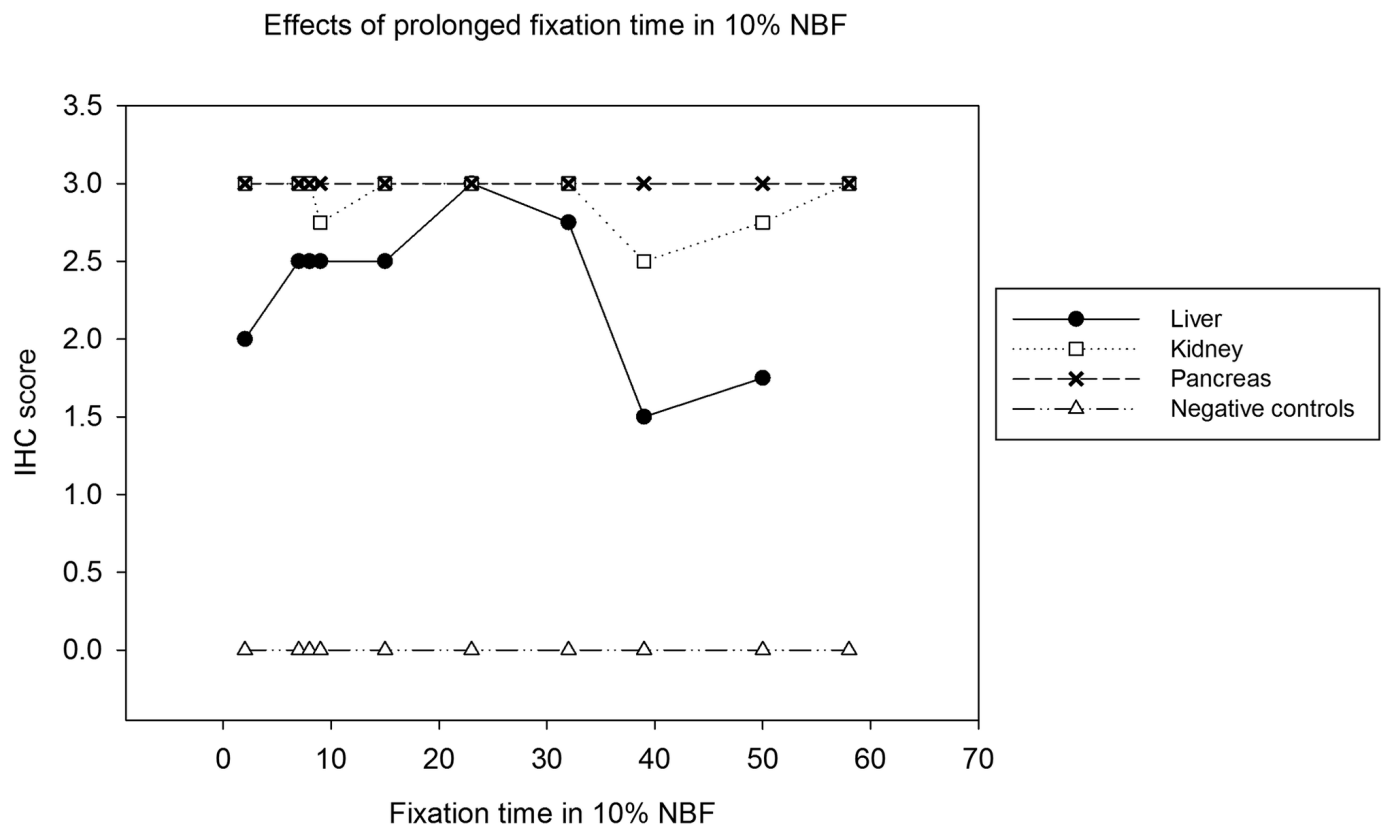
Mean IHC score of liver, kidney, and pancreas over fixation time in 10% NBF.

Ten blocks (Block 1 to 10), each containing liver, kidney, and pancreas that were fixed for a specific length of time (48 hours to 58 days), were used in this study. Three slides were made from each block, two stained with anti-IBDP MAB, and one stained with non-specific mouse antibody as negative control. The inclusion bodies within the embedded tissue remained detectable with the anti-IBDP MAB up to 58 days following initial fixation (Table 3). The staining intensity of inclusion bodies in pancreas was consistently high (IHC score 3) throughout all tested fixation time periods. The staining intensity of inclusion bodies in liver and kidney remained high (IHC score 2-3) up to 32 days following fixation. Some uneven staining (IHC score 2 or 3) were observed in liver and kidney beyond 8 days of fixation. More severe unevenness in staining intensity were observed in the liver (IHC score 1 or 2) and kidney (IHC score 0 or 2 or 3) fixed for 39 days and 50 days. In kidney fixed for 50 days, there were some areas where the inclusion bodies were unstained. In all negative control slides, no staining of the inclusion bodies was observed. Overall, prolonged fixation up to 58 days did not affect the IHC staining in pancreas, but in kidney an uneven staining pattern was observed in samples fixed for 9 days, 39 days, and 50 days. In liver, the staining tended to be less intense and more inconsistent compared to the staining in kidney and pancreas (Figure 6).

**Table 3:** IHC scores of IBD positive tissues fixed in 10% NBF over different time period.

**Block number**		**1**	**2**	**3**	**4**	**5**	**6**	**7**	**8**	**9**	**10**
**Fixation Time (day**)		**2 d**	**7 d**	**8 d**	**9 d**	**15 d**	**23 d**	**32 d**	**39 d**	**50 d**	**58 d**
Slide 1	Liver	2	2	2,3	3	2,3	3	2,3	1,2	1,2	N/A
	Kidney	3	3	3	3	3	3	3	2,3	2,3*	3
	Pancreas	3	3	3	3	3	3	3	3	3	3
Slide 2	Liver	2	3	2,3	2	2,3	3	3	1,2	2	N/A
	Kidney	3	3	3	2,3	3	3	3	2,3	3	3
	Pancreas	3	3	3	3	3	3	3	3	3	3
Slide 3	Liver	0	0	0	0	0	0	0	0	0	N/A
	Kidney	0	0	0	0	0	0	0	0	0	0
	Pancreas	0	0	0	0	0	0	0	0	0	0

The IHC score was not uniformed in some samples, in which two scores were given.

* In some areas the inclusion bodies were not stained by anti-IBDP MAB. N/A: sample not available.

**Figure 7:**
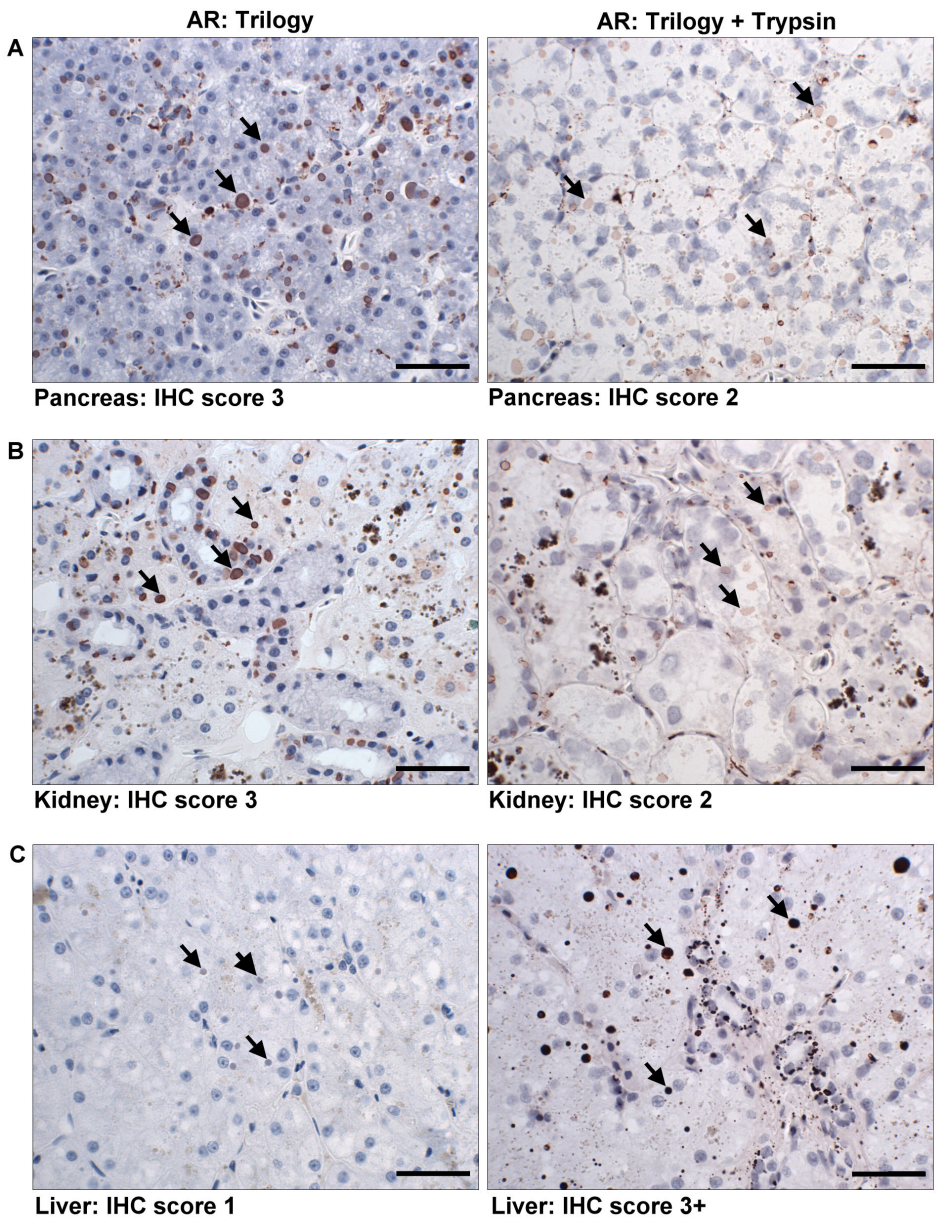
Differences in IHC staining between Trilogy and double AR treatment on prolong fixed tissues. Tissue fixed up to 50 days (Block 9) in 10% NBF was stained with standardized Trilogy AR treatment (left) and double AR treatment (right). The inclusion bodies are indicated by arrows. Bar = 40 µm. A. In pancreas, the staining of inclusion bodies decreased when double AR was used. B. In kidney, the staining of inclusion bodies decreased when double AR was used. C. In liver, the staining of inclusion bodies improved when double AR was used.

The double AR treatment restored the reactivity of anti-IBDP MAB in liver up to 50 days of fixation in 10% NBF (Block 9), and the uneven staining pattern was not observed. When double AR treatment was used in staining liver in Block 8 and 9 (Table 3), the inclusion bodies stained dark red and the intensity was stronger (IHC score 3+)(Figure 7A). Unfortunately, the double AR treatment was judged to be harsher on tissues, which resulted in loss of cellular structural detail. In kidney and pancreas, double AR treatment reduced the staining of inclusion bodies (Figure 7B and C). However, to ensure that insufficient AR was not responsible for failure to stain with IHC, all blocks that stained negative with IHC (including the IBD- cases) were retested with another run using the double AR treatment.

#### Effects of storage time in paraffin

Paraffin embedded tissues of 60 IBD+ and 34 IBD- boa constrictors collected from 1990-2011were tested using IHC staining. Paraffin embedded tissues were classified into one of two groups based on the time period they were collected (Group1:1990-2000 versus Group 2: 2001-2011). The sensitivity of the IHC test was similar between Group 1 (24/29 or 83%) and Group 2 (26/31 or 84%) (p = 0.99) (Table 4). Similarly, the specificity of the IHC test was similar between Group 1 (15/15 or 100%) and Group 2 (18/19 or 95%) (p = 0.99) (Table 5). This suggested that the duration of storage in paraffin blocks did not significantly affect the performance of MAB in IHC staining.

**Table 4:** Sensitivity of IHC test for samples classified as positive by the H&E test that were stored during 1990-2000 or 2001-2011.

	**Group 1 (1990-2000) H&E +**	**Group 2 (2001-2011) H&E +**
**IHC +**	24 (83%)	26 (84%)
**IHC -**	5 (17%)	5 (16%)
**Sample size (%)**	**29 (100%)**	**31 (100%)**

**Table 5:** Specificity of IHC test for samples classified as negative by the H&E test that were stored during 1990-2000 or 2001-2011.

	**Group 1 (1990-2000) H&E -**	**Group 2 (2001-2011) H&E -**
**IHC +**	0 (0%)	1 (5%)
**IHC -**	15 (100%)	18 (95%)
**Sample size (%)**	**15 (100%)**	**19 (100%)**

#### Species cross-reactivity

The reactivity of the anti-IBDP MAB was tested on paraffin embedded tissues of the following 15 IBD+ non-boa constrictors: annulated tree boas (n=3), ball pythons (n=4), carpet pythons (n=2), emerald tree boa (n=1), palm viper (n=1), and rainbow boas (n=4) (Table 6). The samples of annulated tree boas [8] and palm viper [4] were obtained from previously reported studies. The anti-IBDP MAB showed species cross-reactivity to IBD inclusion bodies in ball pythons (1/4) and carpet pythons (2/2). 

**Table 6:** List of IBD positive non-boa constrictors tested with anti-IBDP MAB.

**Species**	**Number of snakes**	**IHC Stain positive**	**IHC Stain negative**
**Annulated tree boas** ^a^	3	0	3
**Ball pythons**	4	1	3
**Carpet pythons**	2	2	0
**Emerald tree boas**	1	0	1
**Palm viper** ^b^	1	0	1
**Rainbow boas**	4	0	4

^a^ Sample of IBD cases reported by Stenglein et al [8].

^b^ Sample of IBD case reported by Raymond et al [4].

#### Antigen cross-reactivity

The reactivity of the anti-IBDP MAB to inclusion bodies of other non-IBD diseases was tested with IHC staining on two previously reported corn snakes [20], a boa constrictor with adenoviral inclusion bodies, and a boa constrictor with pox-virus-like inclusion bodies. No cross-reactivity of the anti-IBDP MAB to the non-IBD inclusion bodies was found in IHC staining.

#### IHC test diagnostic performance

Overall, 60 samples from boa constrictors classified as IBD+ and 34 as IBD- based on H&E staining were tested with IHC staining (Table 7). The sensitivity of the IHC test was 50/60 or 83% (95% CI = 76%, 91%). The specificity of the IHC test was 33/34 or 97% (94%, 100%). Interestingly, out of 34 IBD- cases, one boa constrictor from 2001 that was suspected to be IBD+ based on the clinical signs, but diagnosed IBD- by the pathologist after examination of the H&E stained sections, was found by IHC staining to have small inclusion bodies in liver and brain. These small inclusion bodies were not recognized with H&E stain. Thus, the one IBD- (H&E stained negative) case was actually IBD+. If included in the positive group, the specificity of anti-IBDP MAB in IHC staining should be 100%. In the current study, the prevalence of the sample set was 64%. Assuming the sensitivity and specificity of the IHC test were 83% and 97%, respectively, the PPV should be 98% and the NPV should be 77%. However, if the prevalence of IBD in captive boa constrictors was 5%, the PPV should be 57% and the NPV should be 99%. Using the same sensitivity and specificity, but a prevalence of 15%, the PPV should increase to 79%, and the NPV should decrease to 96% (Table 8).

**Table 7:** Overall performance characteristics of the IHC test in boa constrictors.

**Overall samples (1990-2011)**				**IHC Test Performance Characteristics (%)**		**Lower 95% CI**	**Upper 95% CI**
	H&E +	H&E -	Total IHC Test results	Sensitivity	83.3	75.8	90.9
IHC +	50	1	51	Specificity	97.1	93.6	100
IHC -	10	33	43	Positive predicted value	98.0	95.2	100
Total H&E test results	60	34	94	Negative predicted value	76.7	68.2	85.3
				False positive	2.9		
				False negative	16.7		

The positive and negative predictive values were calculated based on the prevalence (64%) of the sample set.

**Table 8:** The relationship between prevalence and the positive and negative predicted values of the IHC test.

	**IHC Test of Sensitivity: 83%, Specificity: 97%**		**IHC Test of Sensitivity: 83%, Specificity: 100%**	
**Hypothetic Prevalence of IBD (n=94)**	**PPV**	**NPV**	**PPV**	**NPV**
5%	57%	99%	100%	99%
15%	79%	96%	100%	96%
64%	98%	77%	100%	77%
80%	98%	58%	100%	59%

PPV: Positive predicted value; NPV: Negative predicted value.

## Discussion

### Insolubility of the Isolated IBDP

The most challenging aspect of this study was the solubilization of IBDP. Insoluble semi-purified inclusion bodies (IB preps), could not be solubilized completely by a wide range of methods that are commonly used for solubilizing protein. This feature is shared by other protein accumulating disorders such as aggregates of Huntingtin protein, a prion-like protein that is seen in Huntington’s disease [21]. Heating at 100°C with combinations of 1.25% SDS and 1.25% beta-mercaptoethanol were used for denaturing insoluble prion [22]. However, the use of a variety of reducing agents and heating were not effective in solubilizing IBDP. In a study by Wozniak et al, IBDP (68 KDa band obtained from liver homogenate) was electro-eluted from the excised gel and used as an immunogen to produce monoclonal antibody against IBDP [10]. However, in their study the insolubility of IBDP was not reported. In the current study, the electro-eluted IBDP derived from the IB prep (68 KDa band obtained from insoluble inclusion bodies) was insoluble and could not be retrieved. The insolubility and the nature for aggregate formation may indicate that IBD shares a similar disease mechanism with other protein aggregate forming diseases.

Another problem caused by the insolubility of IBDP was the difficulty in estimating the protein concentration by Bradford Protein Assay for the use in ELISA and western blots. Bradford Protein Assay is commonly used to estimate protein concentration in solutions by comparing the coloration of protein binding dyes in an unknown sample to a set of standard proteins with known concentrations [23]. However, high concentrations of detergents in the sample interfered with the coloration of the assay and resulted in inaccurate estimation of the protein concentration. We found that for IBDP, determining the volume needed for a sufficient reaction in each assay provided better assay performance than solely based on the estimated protein concentration of the preparation. 

To ensure that the best IBDP reactive MAB with the highest specificity was selected, IB preps derived from different tissues (liver and kidney) and also from different boa constrictors were used as antigen for antibody screening. When screening the mass cultures, only those that reacted with all IB preps were selected to avoid choosing an antibody that would non-specifically react to contaminants in liver or kidney. The specificity of the clones was confirmed by ELISA, western blots, immuno-TEM and IHC staining, which ensured the antibody selected reacted to the 68 KDa protein and the inclusion bodies in situ with minimal background staining. Based on these findings, the IB preps were of sufficient purity to achieve the objectives of this study.

### Factors to Consider for IHC Staining

This study was designed to validate an anti-IBDP MAB for use in IHC staining to diagnose IBD in boa constrictors. Boa constrictors were considered the standard species, for the following reasons: 1. The anti-IBDP MAB was produced using liver tissue of a boa constrictor; 2. Within the sample repository, the majority of the samples were from boa constrictors; 3. IBD in boa constrictors was more frequently diagnosed than in other species. The outcome of our antibody validation studies designed to evaluate factors that could potentially affect the IHC staining are discussed below.

#### Fixation time in formalin

For immuno-detection purposes, although there is no agreement on the exact fixation time, it is generally recommended that tissues should be fixed for a minimum of 12-24 hours [24]. Prolonged fixation in formalin has been considered a limiting factor for IHC staining, because antigenic epitopes can be masked by cross-linking during formalin fixation [25]. Following fixation in 10% NBF for over 5-8 days, IHC staining intensity may decrease for some antigens, and fixation of 14-36 days or more may result in a further decline or loss in antigen staining by IHC [25-28]. In the current study, 48 hours was considered the standard fixation time in the two different laboratories that were used. However, when multiple tissues from a snake with IBD were fixed in 10% NBF for time periods up to 58 days kidney and pancreas retained moderate to strong reactivity with anti-IBDP MAB, whereas for liver, reactivity diminished following 32 days of fixation (Figure 6). This finding corresponds to the result of several other studies [24,25,27,28], in which prolonged fixation adversely impacted staining quality and intensity.

#### Tissue type

In the standardized staining condition using Trilogy AR treatment, the staining of liver was assessed to be less intense compared to the staining of pancreas and kidney regardless of the fixation time. In liver, the inclusion bodies located at the margin of the tissue stained more intensively then the inclusion bodies located near the center of the tissue section. Similar staining patterns were observed in the study of Webster et al., which was attributed to differences in the rate of formalin penetration [25]. Possibly differences in pH among different tissues or other biochemical differences affected the rate and efficiency of formalin infiltration. Further, findings in this study suggested that the outcome of IHC staining following extended fixation time is tissue dependent. It has been discussed that many antibodies have variable immune-reactivity among different tissue types [25] and that the validation should be performed on the tissue types routinely collected for making a diagnosis [15,24,25]. In IBD affected snakes, the inclusion bodies were most commonly observed in liver, kidney, and pancreas, which we selected as the standard tissues to be validated for IHC test. Although, the IHC test performed more consistently in kidney and pancreas, the liver is often preferable for sampling since it is larger tissue and more accessible to biopsy compared to the pancreas and kidney. However, extended fixation time in 10% NBF should be avoided if liver samples are collected for the IHC test.

#### Selection of AR treatment

Antigen retrieval for IHC staining was to improve the binding of the antibodies to the antigen by breaking down the cross-linked proteins formed through formalin fixation. This can be a critical step, especially when tissues are fixed or remain in fixatives for prolonged periods of time [24,28,29]. In most antigens with proper AR treatment, a moderate staining intensity could still be obtained in tissues fixed more than 7 weeks [25,27]. Improved staining intensity was observed in double AR treated liver that was previously fixed for a prolonged period of time. But when double AR treatment was used, the structural details of the stained tissues were lost due to the harsh treatment. Thus, the standard AR using Trilogy should be considered as a routine procedure, but if the staining in liver is not satisfactory, then double AR can be used. However, increased non-specific background staining in the connective tissues was observed in some samples.

During the process of standardization, we found that the same tissue sample processed and embedded in two laboratories (Lab 1 and Lab 2) had different requirements for AR (Table S2). In addition, of 12 boa constrictors (9 IBD+ and 3 IBD-) that came from a snake collection obtained in 2007, 3 IBD+ boas stained IHC+ only with double AR treatment (Figure S1). It has been reported that variables in tissue processing may result in different IHC staining intensity [28]. It is possible that slight differences during tissue processing, such as, temperature, incubation time, and the source of the reagents accounted for these differences (Table S1). 

### IHC Test Diagnostic Performance Evaluation

In this study, the diagnostic performance of the IHC test was considered acceptable with the sensitivity of 83% and specificity of 97%. The higher false negative value (10/60 or 16.7%) of the IHC test estimated by this study was affected by 10 IHC- cases that were previously diagnosed as IBD+. When the H&E stained sections of the 10 cases were re-examined, the characteristic eosinophilic intracytoplasmic inclusion bodies were not found in 5 of these cases. Thus, we judged the diagnosis of these 5 cases as ‘questionable’. Four of the questionable cases were diagnosed during 1990-2000 (3 were reported in 1995), which was approximately when IBD was being diagnosed more commonly in boa constrictors in the US. This also suggested that a false positive diagnosis of IBD can likely be made by a pathologist having limited experience diagnosing IBD. The limitation of using H&E stained sections for diagnosing IBD is the inability of this method to accurately distinguish IBD inclusion bodies from other types of eosinophilic intracytoplasmic inclusion bodies. Similar problems were described in another IBD study using H&E staining [14]. Regardless of the questionable cases, with the IHC test, our anti-IBDP MAB did not react with inclusion bodies in 5 out of 60 (or 8%) IBD+ boas. This suggests that there may be differences in protein composition and or structure within IBD inclusion bodies among different IBD+ boas to affect reactivity with our MAB. This is supported by recent studies in which IBD inclusion bodies were found to consist of nucleoprotein of arena-like viruses having multiple variants [8,13,14]. There were sufficient differences of the nucleoprotein among different strains that antibody produced against the nucleoprotein of one strain may not cross-react with all strains [8,14]. Therefore, in making a diagnosis of IBD, the results of both H&E and IHC staining should be considered. 

Although the estimated specificity of the IHC test was very high (97%), the calculated false positive value (1/34 or 2.9%) may have been overestimated. The one case judged to be IBD- with H&E staining, was clearly IHC+ in liver and brain. Apparently, due to the small size of inclusions they were missed in the original examination of H&E stained tissue sections. Previous studies have shown that IBD inclusion bodies start as small and inconspicuous intracytoplasmic bodies that increase in size over time [10]. In this study, the result of the immuno-TEM labeling also demonstrated the high specificity of the anti-IBDP MAB in detecting small inclusion bodies (Figure 4A). If we add this case to the group of IBD+ boas, the specificity of the IHC test using anti-IBDP MAB should be 100%. 

Ideally, in order to use the IHC test for diagnosis and control of IBD in populations of boa constrictors, the disease burden (prevalence) in the populations of interest should be known since prevalence will affect PPV and NPV of the test (Table 8). In the current study, the prevalence of the sample set was 64%, and with an IHC test sensitivity and specificity of 83% and 97%, respectively, the PPV should be 98% and the NPV should be 77%. However, if the prevalence increases or decreases in a population, PPV and NPV will also change. For instance, in a population with 5% prevalence of IBD, the PPV of the IHC test should predict disease in 57% of boa constrictors. If the prevalence of IBD is 15%, the PPV of the IHC test should predict disease in 79% of boa constrictors. In addition, if the prevalence of disease was 5% to 15%, the NPV of the IHC test should accurately identify 99% to 96%, respectively, of boa constrictors that were free of IBD. If the IHC test had a specificity of 100%, an IHC+ test result should accurately diagnose IBD independent of the prevalence of the population being tested (Table 8). 

### Cross Reactivity among Non-Boa Constrictors

Of the total number of cases available for this project, the number of IBD+ non-boa constrictors was limited. Throughout 1990 to 2011, only 15 (6 species) cases were found in UF and collaborating laboratories, with, only 2 carpet pythons and 1 ball python staining IHC+. Even when double AR was used, the other 12 cases remained unstained. As with boa constrictors, different species of snakes may be infected with different ‘strains’ of the causative agent of IBD. Interestingly, the two species in our study, in which the anti-IBDP MAB cross-reacted were more distantly related to boa constrictors than other more closely related species tested, such as, rainbow boas, annulated tree boas, and emerald tree boas. Nevertheless, the use of anti-IBDP MAB in diagnosing IBD in boa constrictors is validated by this study and will serve as a powerful tool for diagnosing this insidious disease.

## Supporting Information

Figure S1 **IHC staining of embedded tissue that strictly required double AR treatment.** The paraffin embedded kidney (left) and liver (right) of an IBD+ boa constrictor were stained with anti-IBDP MAB using standard Trilogy treatment and double AR treatment. In this sample, the inclusion bodies (black arrows) were stained only using double AR treatment. Yellow arrow heads showed the pigmented granules in the kidney, and yellow arrows showed the pigmented macrophage in the liver. Bar = 40 µm. A. The IHC staining of liver and kidney using standard Trilogy treatment. The inclusion bodies were not stained by anti-IBDP MAB. B. The IHC staining of liver and kidney using double AR treatment. The inclusion bodies were stained with high intensity.
(TIF)

Table S1 **The tissue processing procedure in two laboratories.**
(PDF)

Table S2 **IHC score of tissues processed by two laboratories using different AR treatments.**
(PDF)
