# Supplementary figures and images for: Immunohistochemical Detection of a Unique Protein within Cells of Snakes Having Inclusion Body Disease, a World-Wide Disease Seen in Members of the Families Boidae and Pythonidae
Source: PLoS One. 2013 Dec 10;8(12):e82916. doi: 10.1371/journal.pone.0082916 (PMC3858296; doi:10.1371/journal.pone.0082916)

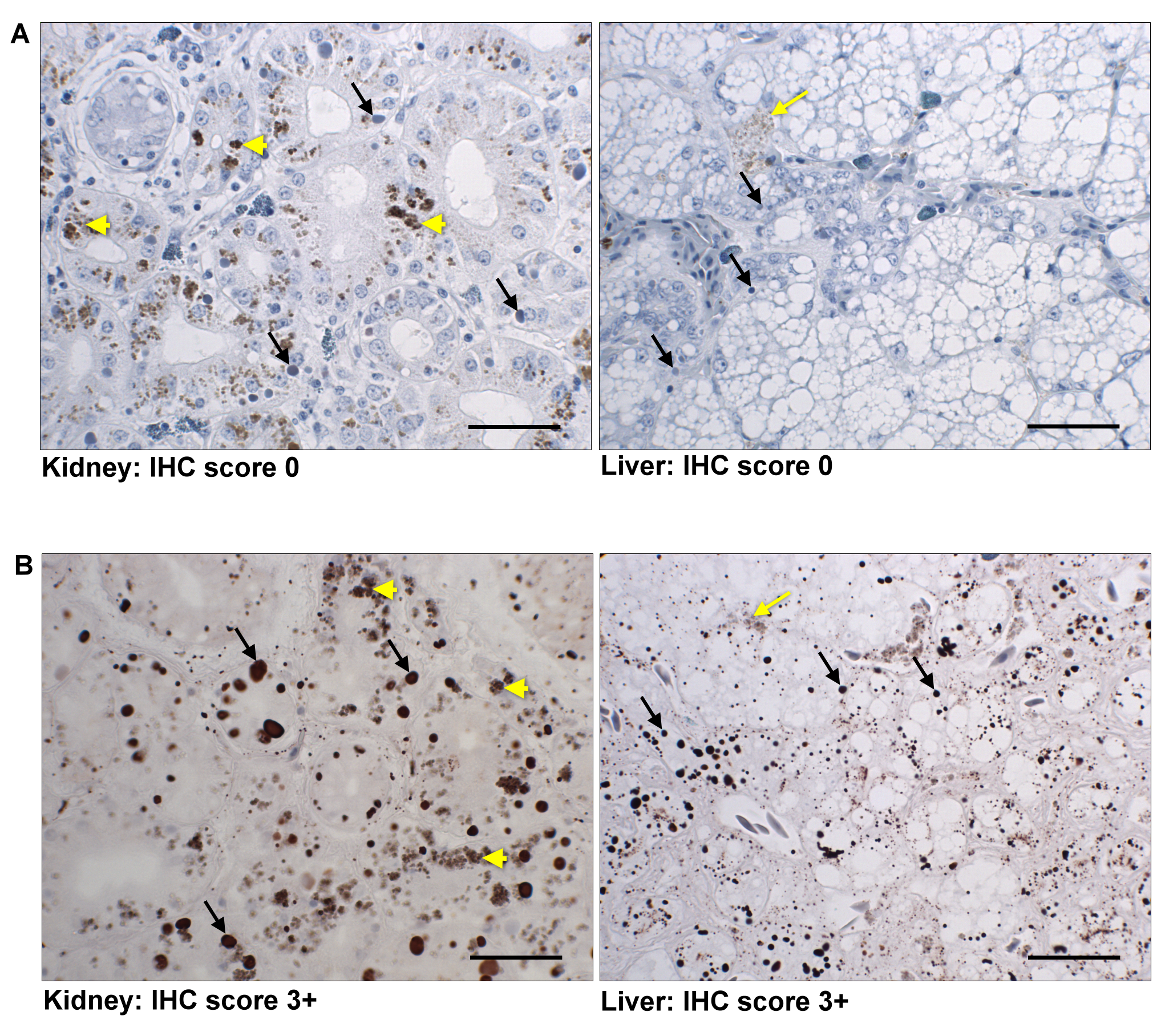

Supplement: Figure S1 — IHC staining of embedded tissue that strictly required double AR treatment. The paraffin embedded kidney (left) and liver (right) of an IBD+ boa constrictor were stained with anti-IBDP MAB using standard Trilogy treatment and double AR treatment. In this sample, the inclusion bodies (black arrows) were stained only using double AR treatment. Yellow arrow heads showed the pigmented granules in the kidney, and yellow arrows showed the pigmented macrophage in the liver. Bar = 40 µm. A. The IHC staining of liver and kidney using standard Trilogy treatment. The inclusion bodies were not stained by anti-IBDP MAB. B. The IHC staining of liver and kidney using double AR treatment. The inclusion bodies were stained with high intensity. (TIF) [file pone.0082916.s001.tif]
