# Supplementary material for: Immunohistochemical Detection of a Unique Protein within Cells of Snakes Having Inclusion Body Disease, a World-Wide Disease Seen in Members of the Families Boidae and Pythonidae
Source: PLoS One. 2013 Dec 10;8(12):e82916. doi: 10.1371/journal.pone.0082916 (PMC3858296; doi:10.1371/journal.pone.0082916)
Supplement: Table S1 — The tissue processing procedure in two laboratories. (PDF) [file pone.0082916.s002.pdf]

Table S1. The tissue processing procedure in two laboratories.

	Lab1 Processor			Lab2 Processor		
Reagent	Incubated Time (sec/run)	Number of runs	Temperature	Incubated Time (sec/run)	Number of runs	Temperature
Formalin	30	2	Ambient	N/A	N/A	N/A
70% EtOH	60	1	30˚C	1	1	Ambient
				20	1	Ambient
80% EtOH	60	1	30˚C	20	1	Ambient
95% EtOH	60	1	30˚C	20	2	Ambient
100% EtOH	60	3	30˚C	20	2	Ambient
Xylene	40	3	30˚C	20	2	Ambient
Paraffin Wax	40	3	60˚C	20	3	60˚C

EtOH: ethanol. N/A: not applicable.
In Lab1, the reagents used were Alcohol (Decon Laboratories), Histology Grade Xylene (Fisher), and Paraplast (Fisher). Formalin was made with 37% Formaldehyde (Fisher), Sodium Phosphate Monobasic anhydrous (MP laboratories), and Sodium Phosphate Dibasic, anhydrous (Fisher).
In Lab2, the reagents used were NBF 10% buffered (Fisher), Ethanol (Fisher), Histology Grade Xylene (Fisher), and Paraffin Type9 (Fisher).
