# Supplementary material for: Immunohistochemical Detection of a Unique Protein within Cells of Snakes Having Inclusion Body Disease, a World-Wide Disease Seen in Members of the Families Boidae and Pythonidae
Source: PLoS One. 2013 Dec 10;8(12):e82916. doi: 10.1371/journal.pone.0082916 (PMC3858296; doi:10.1371/journal.pone.0082916)
Supplement: Table S2 — IHC score of tissues processed by two laboratories using different AR treatments. (PDF) [file pone.0082916.s003.pdf]

Table S2. IHC score of tissues processed by two laboratories using different AR treatments.

Tissue embedded	Primary antibody	Sample origin	AR reagents					
			No AR	Trypsin	Trilogy	Dako pH9.0	Dako pH6.0	Citrate
Lab 1	MAB	08-122 Pancreas	2	3	3	2	2	2
Lab 2	MAB	08-122 Pancreas	0	0	3	2	2	2
Lab 2	MAB	08-76 Pancreas	1	1	3	2	3	3
Lab 1	None specific mouse IgG	08-122 Pancreas	0	0	0	0	0	0

MAB: monoclonal anti-IBDP antibody.

Boa 08-76 and 08-122 were two IBD+ boa constrictors, the MAB was produced against the inclusion bodies isolated from boa 08-76.
